# miR-29a-5p Regulates the Proliferation, Invasion, and Migration of Gliomas by Targeting DHRS4

**DOI:** 10.3389/fonc.2020.01772

**Published:** 2020-09-10

**Authors:** Yong Dai, Zhenhua Chen, Wei Zhao, Gang Cai, Zhifeng Wang, Xuejiang Wang, Hongkang Hu, Yi Zhang

**Affiliations:** ^1^Department of Neurosurgery, Second Affiliated Hospital of Nantong University, Nantong, China; ^2^Department of Neurosurgery, Changzheng Hospital, Second Military Medical University, Shanghai, China

**Keywords:** gliomas, miR-29a-5p, DHRS4, proliferation, invasion, migration

## Abstract

Gliomas are the most common malignant primary brain tumors in adults and exhibit a spectrum of aberrantly aggressive phenotypes. MicroRNAs (miRNAs) play a regulatory role in various cancers, including gliomas; however, their specific roles and mechanisms have not been fully investigated. Studies have indicated that miR-29a is a tumor-suppressive miRNA, but the data are limited. In this study, we investigated the role of miR-29a-5p in glioma and further explored its underlying mechanisms. On the basis of bioinformatics, dehydrogenase/reductase 4 (DHRS4) was considered a potential target of miR-29a-5p and was also found to be highly expressed in gliomas in our experiments. Moreover, with a luciferase reporter assay, DHRS4 was found to be a target gene of miR-29a-5p and to be correlated with glioma proliferation, invasion, and migration in our *in vivo* and *in vitro* experiments. Simultaneously, we observed that the knockdown of DHRS4 rescued the downregulation of glioma proliferation, invasion, and migration caused by treatment with a mir-29a-5p inhibitor. The present findings demonstrate that miR-29a-5p suppresses cell proliferation, invasion, and migration by targeting DHRS4, and DHRS4 may be a potential new oncogene and prognostic factor in gliomas.

## Introduction

Gliomas are the most common primary tumors of the nervous system in adults (1). Malignant gliomas, especially glioblastomas, are characterized by rapid invasion and growth and are associated with poor clinical prognosis (2, 3). Despite advances in chemotherapy and radiotherapy, most patients succumb to the disease within 5 years (4). Therefore, the identification of prognostic biomarkers and therapeutic targets for the treatment of gliomas is essential for the diagnosis, treatment, and prognostic evaluation of gliomas (5, 6).

MicroRNAs (miRNAs) are endogenous, single-stranded, non-coding, small RNAs comprising approximately 22 nucleotides (7). miRNAs were first discovered in 1993 (8) and are involved in the invasion and inhibition of many tumors (9, 10). In addition, miRNAs are associated with the biological behavior of cancers and may serve as valuable prognostic biomarkers (11, 12). The miR-29 family has three closely related members, namely, miR-29a, miR-29b, and miR-29c (13). miR-29a, miR-29b, and miR-29c are closely related to the inhibition and downregulation of colorectal cancer (14), lung cancer (15), and hepatocellular carcinoma (16) and suppress invasion in glioma by targeting CDC42 (17). Research has shown that miR-29a is a multitarget regulator and is involved in various aspects of cancer cell behavior, such as proliferation, apoptosis, and invasion (18). Moreover, miR-29a is expressed in aggressive glioblastoma subclasses, and its expression correlates with short patient survival (19). miR-29a-3p expression is negatively correlated with PROM1 expression in laryngocarcinoma tissues (20). However, whether miR-29a-5p regulates function in glioma remains unknown and needs to be examined further.

In this study, we confirmed that mir29a-5p acts as a tumor suppressor in gliomas. Although several targets of miR-29a have been identified (21, 22), using TargetScan and miRanda analysis, we focused on dehydrogenase/reductase 4 (DHRS4) and explored its role as a target of mir29a-5p. Furthermore, we clarified the role of DHRS4 in glioma through *in vitro* and *in vivo* experiments. This study provides insight into the molecular mechanisms underlying the proliferation, invasion, and migration of gliomas and may provide a new direction for molecular targeted therapy.

## Materials and Methods

### Patient Tissue Samples

Human glioma tissue specimens (confirmed by histological diagnosis) were obtained from 20 patients who underwent hepatectomy at Changzheng Hospital (Shanghai, China) between 2010 and 2013. Normal brain tissue samples obtained from six patients with severe head trauma for which partial resection of the normal brain tissues was required for decompression were used as controls. Prior to brain resection, no treatments, including radiotherapy and chemotherapy, had been carried out in these patients. Each patient provided written informed consent before participating in the study, and the use of the tumor samples for research was approved by the Specialty Committee on the Ethics of Biomedicine Research of the Second Military Medical University (Shanghai, China). All tumors were classified according to the World Health Organization criteria for tumors of the central nervous system and were immediately frozen after surgery until analysis. The treatment was carried out according to the National Comprehensive Cancer Network guidelines in all patients with glioma included in this study. Clinical follow-up was available for all patients.

### Cell Culture

Rat glioblastoma cell lines and the human glioblastoma cell lines C6 and U87 were obtained from laboratory preservation. Astrocytes were extracted from 1- to 2-day-old postnatal Sprague–Dawley rats. All cell lines were cultured in Dulbecco modified eagle medium (DMEM; Gibco, Waltham, MA, United States)^1^ supplemented with 10% fetal bovine serum (FBS) (Gibco) and 1% penicillin-streptomycin (Gibco). Cells were cultured in a humidified atmosphere at 37°C under 5% CO_2_.

### Real-Time Polymerase Chain Reaction

Total RNA enriched for miRNA was extracted from C6 and U87 cell lines with TRIzol (Invitrogen, Carlsbad, CA, United States) (see text footnote 1). cDNA was prepared using 1 μg of total RNA from each sample (SuperScript III First-Strand Synthesis SuperMix; Invitrogen). A miR-29a-5p–specific real-time polymerase chain reaction (RT-PCR) primer was purchased from Applied Biosystems. Six nanograms of cDNA was used for real-time PCR analysis in a final reaction volume of 20 μL. The samples were analyzed in triplicate using the StepOnePlus^TM^ RT-PCR system (Applied Biosystems, Foster City, CA, United States)^2^, and statistical analysis was performed using the *t* test.

### Western Blot Analysis

Protein was extracted from cells using RIPA buffer (Beyotime, China) supplemented with 1 mM PMSF (Beyotime). The protein concentration was measured by the BCA protein assay kit (Beyotime), and protein was transferred to polyvinylidene difluoride membranes (Millipore, Bedford, MA, United States). The membranes were blocked in 5% non-fat dry milk and then incubated with primary antibodies overnight at 4°C, followed by incubation with horseradish peroxidase–conjugated secondary antibodies in Tris-buffered saline for 2 h at room temperature. Finally, protein bands were visualized using an enhanced chemiluminescence detection system (Pierce, Thermo Fisher Scientific).

### *In vitro* miRNA Mimics/Inhibitor and Cell Transfection

Oligonucleotides encoding miR-29a-5p mimics or inhibitors and scramble controls were purchased from Ribo Life Science (Suzhou, China). Cells were transfected using Lipofectamine RNAiMAX reagent purchased from Invitrogen following the manufacturer’s guidelines. The concentration of mi-29a-5p mimics was 50 nM in C6 and U87 cells, whereas the concentration of mi-29a-5p inhibitor was 100 nM.

### Target Prediction and Dual-Luciferase Reporter Assays

Candidate targets of miR-29a-5p were predicted using the free online tool TargetScan^3^. The DHRS4 3’-untranslated region (3’-UTR) containing the predicted wild-type binding site for miR-29a-5p was amplified and cloned into the pmirGLO vector (Promega Corporation). The DHRS4 3’-UTR containing the mutant binding site for miR-29a-5p was subcloned by site-directed mutagenesis. At 48 h after transfection with miR-29a-5p mimics, luciferase activity was detected using a dual-luciferase reporter assay system (Promega Corporation) and normalized to Renilla activity.

### Cell Proliferation Assay

Cell proliferation was measured using a CCK-8 Assay Kit (Dojindo Corp.). Cells (2,500) were plated into each well of a 96-well plate and transfected with miR-29a-5p mimics or inhibitors. On the day of harvest, 10 μL CCK-8 was added to 90 μL of culture medium. The cells were incubated for 2 h at 37°C, and the optical density was measured at 450 nm. Three independent experiments were performed. Cell proliferation ability was also assessed using the 5−ethynyl−2’−deoxyuridine (EdU) proliferation assay. At 24 h after transfection, treated cells were resuspended and seeded in 96−well plates at a density of 1 × 104 cells per well and cultured for 24 h. After three washes in phosphate-buffered saline (PBS), the cells were incubated for 4 h in serum−free DMEM supplemented with 50 μM EdU (Guangzhou Ribobio Biotechnology, Co., Ltd., Guangzhou, China). Then, the cells were fixed with 4% polyformaldehyde in PBS at room temperature for 30 min. Finally, the cells were incubated with Apollo staining solution and Hoechst 33342 for 30 min. The percentage of EdU−positive cells relative to the total number of cells was considered to represent the proliferation index. A fluorescence microscope (Olympus Corp.) was used to acquire images, and five random fields (magnification ×100) were selected to evaluate the proliferation rate.

### Transwell Assays

The cell invasion assay was performed with 24-well Invasion Transwell (Corning United States) according to the manufacturer’s guidelines. Transwell chambers were coated with BD Matrigel matrix (Corning), as indicated by the manufacturer, and 3 × 10^4^ U87 and C6 cells were seeded on the BD Matrigel in the upper chamber with serum-free medium. The lower chamber was filled with complete culture medium containing 10% FBS. For the Transwell migration assays, 3 × 10^4^ U87 and C6 cells were suspended in medium without serum and placed on the top side of a polycarbonate Transwell filter without BD Matrigel matrix in the upper chamber of a 24-well Transwell, and medium without serum was used in the lower chamber. After 36 h, the invaded and migrated cells on the lower membrane surface were washed twice with PBS buffer and fixed in 4% paraformaldehyde for 30 min, stained with 0.5% crystal violet solution. A fluorescence microscope by counting of six high-powered fields in the center of each well was selected to evaluate the invasion rate. The% invasion of each group using the following formula: % invasion = experimental group/control group × 100%.


$$\mathrm{Experimental}\,\mathrm{group}\,{\text{n}}_{\text{EX}}/{\text{N}}_{\text{EX}}\,\mathrm{Control}\,\mathrm{group}:n_{\text{Ctrl}}/{\text{N}}_{\text{Ctrl}}$$

n_*EX*_ is the number of cells in each field of view in the lower chamber by the experimental group.

N_*EX*_ is the number of cells in each field of view in the upper chamber by the experimental group.

n_*Ctrl*_ is the number of cells in each field of view in the lower chamber by the control group.

N_*Ctrl*_ is the number of cells in each field of view in the upper chamber by the control group.

### Cell Wound Healing Assays

Cell motility capacity was determined with wound healing assays. The transfected U87 and C6 cells were seeded on six-well plates and grown until reaching confluence. Then, a single scratch wound was generated with a 1,000 μL pipette tip. After being gently washed with PBS twice, the wounded cell monolayer was allowed to heal in serum-free medium. The scratch wounds were photographed with an inverted microscope and digital camera, and quantification was performed with ImageJ software. The results are expressed as a percentage of wound closure by setting the initial scratch width as 100%.

### Cell Cycle Analysis

Cell proliferation was measured by cell cycle analysis. The cells were collected by centrifugation; after discarding the supernatant, the cells were washed twice with prechilled PBS. Then precooled 70% ethanol was added and fixed overnight at 4°C. After centrifugation, the cells were collected again. After washing once with PBS, the cells were added to 500 μL PBS containing 50 μg/mL ethidium bromide (PI), 100 μg/mL RNase A, and 0.2% Triton X-100. Finally, on machine testing, it generally counted 2–3 million cells; the results were analyzed with the cell cycle fitting software ModFit.

### *In vitro* siRNA Transfection

Cholesterol-modified small-interfering RNA for DHRS4 (si-DHRS4) and control siRNA (si-Con) were obtained commercially (RiboBio, Guangzhou, China)^4^. The U87 and C6 cells were removed, digested with trypsin, and seeded into 96-well plates at a density of 104 cells to achieve a transfection density of 30–50%. Next, 0.25 μL of 20 μM siRNA stock solution (RiboBio) was diluted with 6 μL of 1 × riboFECTTMCP Buffer (RiboBio) and mixed gently. Next, 0.6 μL of riboFECTTM CP Reagent (RiboBio) was added and mixed by gentle pipetting and then incubated at room temperature for 0–15 min. Finally, riboFECTTMCP Transfection Complex was added to the appropriate amount of medium, and the culture plate was incubated at 37°C for 24 to 96 h.

### *In vivo* siRNA Transfection

DHRS4 (si-DHRS4) and control siRNA (si-Con) were obtained commercially, as mentioned previously. We first used U87 cells to establish a xenograft model in the backs of nude mice. Then 10 nmol siRNA was diluted in 50 μL RNase-free water and added to 225 μL PBS (RiboBio), When the tumor grew to a size of 5 mm × 5 mm, a 50-μL dilution was collected for multiple injections, and injection was repeated every 3 days for 2–4 weeks. Next, we calculated the tumor weight every day and used section Ki67 staining, RT-PCR, and Western blotting to detect the inhibitory effect.

### Statistical Analysis

All data are presented as the mean ± standard deviation (SD) of at least three independent experiments. Most statistical analyses were made using a one-way analysis of variance, followed by a Dunnett multiple-comparisons test. *p* < 0.05 was considered to be statistically significant.

## Results

### miR-29-5p Expression in Glioma Cell Lines

**FIGURE 1:**
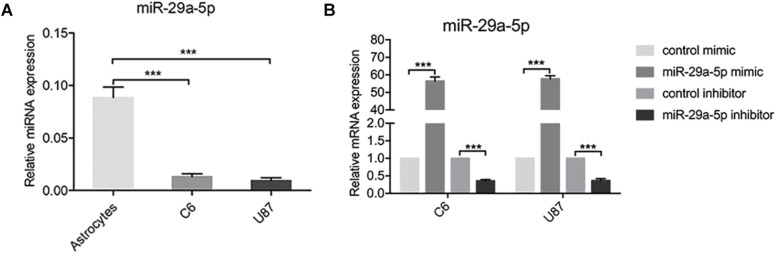
miR-29-5p expression in glioma cell lines. **(A)** miR-29a-5p expression was quantified by RT-PCR analysis. miR-29a-5p expression was significantly lower in both U87 and U251 cells than in the normal astrocytes, ***p < 0.001. **(B)** Quantitative RT-PCR analyses of miR-29a-5p in C6 and U87 cells with miR-29a-5p mimics and inhibitor or the control cells, ***p < 0.001.

To examine the role of miR-29-5p in the progression of glioma, the expression levels of miR-29-5p were measured in astrocytes and C6 and U87 cells by RT-PCR. miR-29a-5p expression was significantly lower in glioma cells than in normal astrocytes (Figure 1A; *p* < 0.001). To further explore the role of mir-29a-5p in glioma cells, chemically synthesized miR-29a-5p mimic and inhibitor were transfected into the U87 and C6 cell lines to upregulate and downregulate miR-29a-5p expression. The transfection efficiency was evaluated via RT-PCR (Figure 1B; *p* < 0.001 for both).

### miR-29a-5p Overexpression Decreases Glioma Cell Proliferation, Invasion, and Migration

**FIGURE 2:**
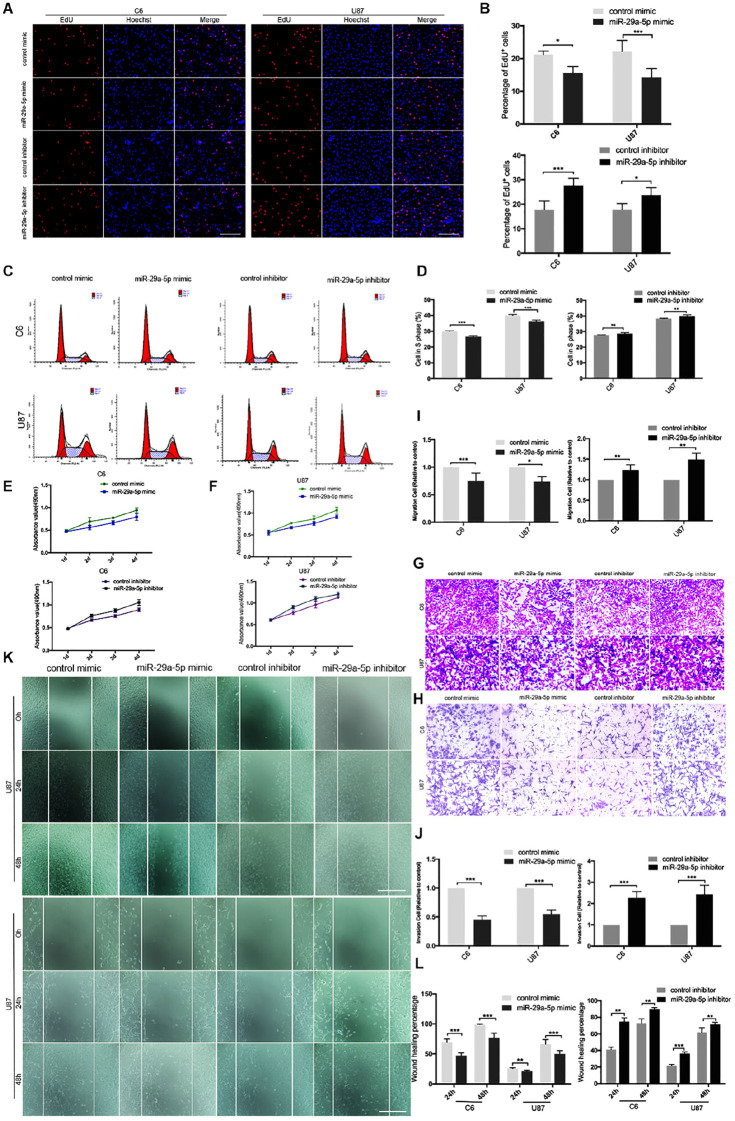
miR-29a-5p overexpression decreases glioma cell proliferation, invasion, and migration. **(A,B)** Fluorescence micrographs (left panels) and data quantification (right panel) from BrdU proliferation assays used to investigate the effects of miR-29a-5p upregulation and downregulation on BrdU (red) incorporation into nuclei (DAPI, blue) in C6 and U87 cells. Data shown in the right panel are mean ± SEM, *p < 0.05, ***p < 0.001, unpaired *t* test. Scale, approximately 100 μm. **(C,D)** Flow cytometry was used to assess the cell cycle distribution of C6 and U87 cells transfected with miR-29a-5p mimics and inhibitor or the control cells for 24 h and stained with PI (left panels). Representative and quantitative results for the S phase are shown (right panel) **p < 0.01, ***p < 0.001. **(E,F)** Cell proliferation was measured by CCK-8 analysis starting 1 day after transfection with miR-29a-5p mimics and inhibitor or the control cells in C6 and U87 cells every day. **(G,I)** Transwell assays of the migration in C6 and U87 cells transfected with miR-29a-5p mimics and miR-29a-5p inhibitor or the control cells, Representative and quantitative results for migration are shown. Columns are the averages of three independent experiments, *p < 0.05, **p < 0.01, ***p < 0.001. **(H,J)** Transwell assays of the invasion in C6 and U87 cells transfected with miR-29a-5p mimics and miR-29a-5p inhibitor or the control cells; invasion of the above cells was quantitatively analyzed. Columns are the averages of three independent experiments, *p < 0.05, **p < 0.01, ***p < 0.001. **(K,L)** Wound healing assays revealing wound closure with miR-29a-5p mimics and miR-29a-5p inhibitor or the control cells in C6 and U87 cells at 0-, 24-, and 48–h timepoints after transfection. Columns are the averages of three independent experiments, **p < 0.01, ***p < 0.001.

First, mRNA expressions of miR29a-5p mimic and miR29a-5p inhibitor in C6 and U87 cells were detected by RT-PCR, respectively (Supplementary Figure S1A; *p* < 0.001). The results of EdU assays and statistical analyses showed that the number of EdU-positive cells was significantly lower in mimic-induced miR-29a-5p–overexpressing C6 and U87 cells than the control cells and increased in miR inhibitor–transfected cells (Figures 2A,B; *p* < 0.05, *p* < 0.001, respectively). Flow cytometric analysis and statistical analyses demonstrated that mimic-induced miR-29-5p overexpression induced cell cycle arrest and decreased the percentage of cells in S phase in both C6 and U87 cells (Figures 2C,D; *p* < 0.01, *p* < 0.001, respectively). The results of the CCK-8 assay demonstrated that mimic-induced miR-29-5p overexpression led to a statistically significant decrease in C6 and U87 glioma cell proliferation compared with the control cells. Conversely, the proliferation rate of C6 and U87 glioma cells treated with the miR-29-5p inhibitor was higher than that of control cells (Figures 2E,F). The effect of miR-29a-5p on migration and invasion was investigated in C6 and U87 cells with Transwell assays. Through statistical analysis, we found that the number of miR-29a-5p–overexpressing C6 and U87 cells penetrating through the Transwell was significantly lower than the control cells and the number of miR inhibitor–transfected cells was higher than the control cells (Figures 2G,H; *p* < 0.05, *p* < 0.01, *p* < 0.001, respectively). The invasion alternation was further confirmed by Transwell assays and yielded similar results to those as above (Figures 2I,J; *p* < 0.05, *p* < 0.01, *p* < 0.001, respectively).

The results of the wound healing assay and statistical analyses demonstrated that miR-29a-5p overexpression significantly inhibited C6 and U87 cell motility (Figures 2K,L; *p* < 0.01, *p* < 0.001, respectively). Taken together, the results above suggested that miR-29a-5p plays a tumor suppressor role in glioma.

### DHRS4 Is the Direct Targeted of miR-29-5p

**FIGURE 3:**
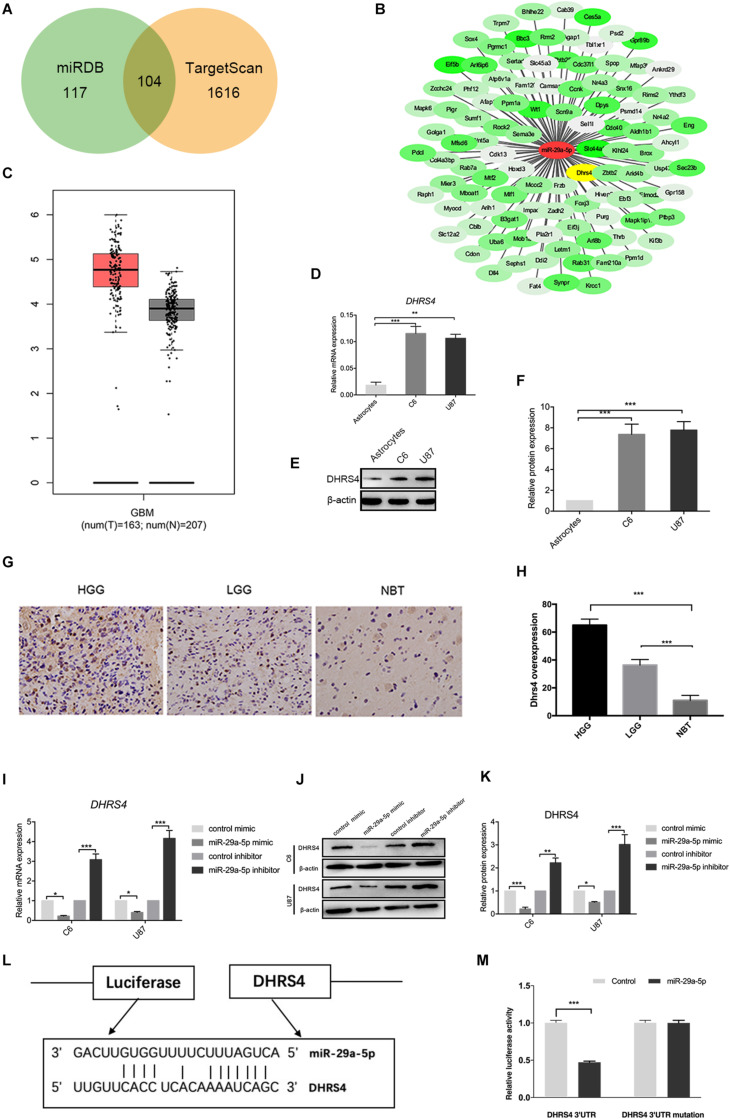
DHRS4 is a direct target of miR-29-5p. **(A,B)** The potential targeted genes, on the basis of the predictions by the two algorithms are shown on the left; the predicted overlapping genes are listed on the right. DHRS4 was within the overlapping area. **(C)** Expression of DHRS4 in tumor (T) specimens and normal (N) brain tissue specimens, as determined by the GEPIA software. **(D)** Quantitative RT-PCR analyses of the DHRS4 mRNA levels were performed between astrocytes and glioma cells. GAPDH was used as a control. Error bars indicate SD, **p < 0.01, ***p < 0.001. **(E,F)** Western blot analyses of the DHRS4 protein levels were performed between astrocytes and glioma cells. Data shown in the right panel are mean ± SEM, ***p < 0.001. **(G,H)** Representative IHC staining for high-grade glioma (HGG), low-grade glioma (LGG), and normal brain tissue (NBT), with detection of DHRS4 expression on the left. Data are shown at right, ***p < 0.001. **(I)** Quantitative RT-PCR analyses of Dhrs4 mRNA levels were performed after treatment of C6 and U87 cells with miR-29a-5p mimics and inhibitor or NC. **(J,K)** Western blot analyses of the DHRS4 protein levels were performed after treatment of U87 cells and C6 cells with miR-29a-5p mimics and inhibitor or NC. Data shown in the right panel are mean ± SEM, *p < 0.05; **p < 0.01 ***p < 0.001 **(L)** The 3’-UTR of the DHRS4 gene contains binding sites for miR-29a-5p, according to bioinformatics analysis. **(M)** miR-29a-5p suppresses the expression of a luciferase reporter gene harboring the 3’-UTR of DHRS4. C6 cells were transiently cotransfected with miR-29a-5p and the indicated luciferase constructs, and luciferase activity was analyzed 48 h later. Data were assessed from three independent experiments, and the *p* values were calculated with a *t* test (***p < 0.001).

Next, we searched for candidate targets of miR-29a-5p. Analysis of miRNA target prediction sites identified 117 genes in miRanda and 1,616 genes in TargetScan as potential targets of miR-29a-5p in glioma cells. Among these genes, 104 showed overlap (Figures 3A,B). DHRS4 was selected for further study because it is highly expressed in glioma, according to GEPIA software (Figure 3C). RT-PCR and Western blot data showed that DHRS4 was expressed at high levels in C6 and U87 cells and at low levels in astrocytes (Figures 3D–F; *p* < 0.01, *p* < 0.001, respectively). The expression level of DHRS4 was also significantly up-regulated in the high-grade glioma tissues compared with normal brain tissues and low-grade glioma tissues, on the basis of immunohistochemical staining (Figures 3G,H; *p* < 0.001). To further explore the relationship between mir-29a-5p and DHRS4, we added miR-29a-5p mimic and inhibitor to C6 and U87 cells. RT-PCR and Western blotting demonstrated that miR-29a-5p overexpression led to lower expression of DHRS4, whereas miR-29a inhibitor caused the opposite result (Figures 3I–K; *p* < 0.05, *p* < 0.01, *p* < 0.001, respectively). To determine whether miR-29a-5p directly interacts with the DHRS4 mRNA 3’-UTR, we used a luciferase reporter system. Cells were cotransfected with miR-29a-5p mimic or control miRNA and DHRS4 3’-UTR-wt or DHRS 3’-UTR-mu. Luciferase assays showed that cotransfection with miR-29a-5p and DHRS4 3’-UTR-wt dramatically decreased luciferase activity, compared with that in the other groups, indicating that the 3’-UTR of DHRS4 was a direct target of miR-29a-5p (Figures 3L,M; *p* < 0.001 for both). Taken together, the results demonstrated that DHRS4 is a direct target of miR-29a-5p in glioma cells and negatively correlates with miR-29a-5p.

### miR29a-5p Rescues DHRS4 Expression *in vitro*

**FIGURE 4:**
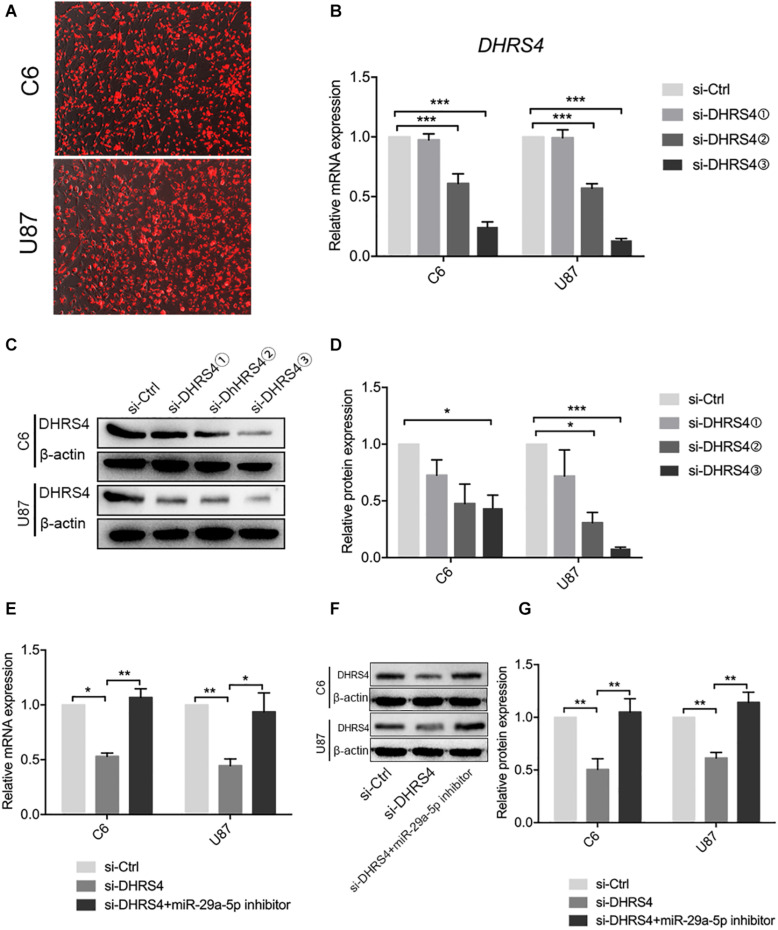
miR29a-5p rescues DHRS4 expression *in vitro*. **(A)** Cy3 expression of DHRS4 after transfection of siRNA. **(B)** Quantitative RT-PCR analyses of the DHRS4 mRNA levels were performed after the treatment of C6 and U87 cells with si-DHRS4 and si-Ctrl. **(C,D)** Western blot analyses of the DHRS4 protein levels were performed after treatment of U87 cells and C6 cells with si-DHRS4 and si-Ctrl. Data shown in the right panel are mean ± SEM, *p < 0.05; **p < 0.01, ***p < 0.001. **(E)** Quantitative RT-PCR analyses of the DHRS4 mRNA levels were performed after the treatment of C6 and U87 cells with si-DHRS4 and cotransfection of miR-29a-5p inhibitor, *p < 0.05; **p < 0.01. **(F,G)** Western blot analyses of the DHRS4 protein levels were performed after treatment of U87 cells and C6 cells with si-DHRS4 and cotransfection of miR-29a-5p inhibitor. Data shown in the right panel are mean ± SEM, *p < 0.05; **p < 0.01.

The above results revealed that DHRS4 is a direct target of miR-29a-5p. We used small interfering RNA (siRNA) to knock down the expression of DHRS4 *in vitro* (Figure 4A). RT-PCR and Western blot data indicated that the mRNA and protein levels of DHRS4 were markedly downregulated in cells treated with si-DHRS4 (Figures 4B–D; *p* < 0.05, *p* < 0.01, *p* < 0.001, respectively). Next, we used miR-29a-5p inhibitor to treat C6/si-DHRS4 and U87/si-DHRS4 cells. RT-PCR and Western blotting revealed that the mRNA and protein levels of DHRS4 showed no marked changes compared with those in si-Ctrl and but showed marked up-regulation compared with those in si-DHRS4 (Figures 4E–G; *p* < 0.05, *p* < 0.01, respectively), thus indicating that downregulation of miR29a-5p levels rescued DHRS4 expression in si-DHRS4 cells.

### Knockdown of DHRS4 Inhibits Glioma Cell Proliferation, Invasion, and Migration

**FIGURE 5:**
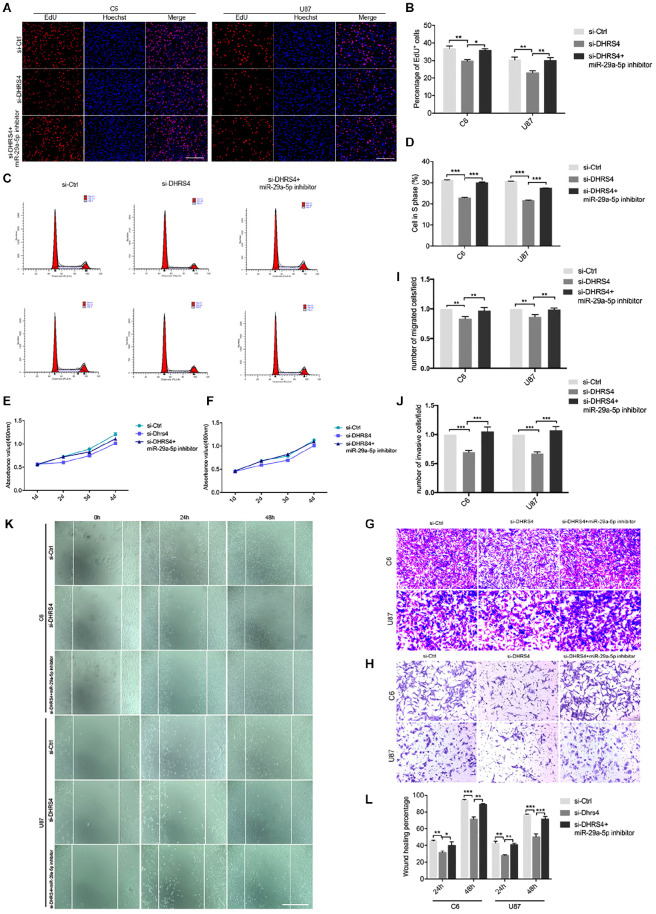
Knockdown of DHRS4 inhibits glioma cell proliferation, invasion, and migration. **(A,B)** Fluorescence micrographs (left panel) and quantification (right panel) of BrdU proliferation assay data for si-Ctrl, si-DHRS4, or si-DHRS4 plus miR-29-5p in C6 and U87 cells. Data shown in the right panel are mean ± SEM, *p < 0.05, **p < 0.01, unpaired *t* test. Scale, approximately 100 μm. **(C,D)** Flow cytometry was used to assess the cell cycle distribution of C6 and U87 cells transfected with si-Ctrl, si-Dhrs4, or si-DHRS4 plus miR-29-5p inhibitor for 24 h and stained with PI (left panels). Representative and quantitative results of S phase are shown (right panel), ***p < 0.001. **(E,F)** Cell proliferation from the above transfected cells was measured with CCK-8 analysis. **(G,I)** Transwell assays of the migration in C6 and U87 cells with si-Ctrl, si-DHRS4, or si-DHRS4 plus miR-29-5p inhibitor. Representative and quantitative results for migration are shown. Columns are the averages of three independent experiments, **p < 0.01. **(H,J)** Transwell assays of the invasion in C6 and U87 cells with si-Ctrl, si-DHRS4, or si-DHRS4 plus miR-29-5p inhibitor. Representative and quantitative results for invasion are shown. Columns are the averages of three independent experiments, ***p < 0.001. **(K,L)** Wound healing assay results revealed wound closure of C6 and U87 with si-Ctrl, si-DHRS4, or si-DHRS4 plus miR-29-5p inhibitor. Columns are the averages of three independent experiments, *p < 0.05; **p < 0.01, ***p < 0.001.

To further evaluate the role of DHRS4 in glioma cells, we performed Edu assays, which showed that the proliferation rate of cells transfected with si-DHRS4 was significantly lower than that of control cells, whereas no changes were observed when cells were cotransfected with si-DHRS4 and miR-29a-5p inhibitor (Figures 5A,B; *p* < 0.05, *p* < 0.01, respectively). Flow cytometry and statistical analyses demonstrated that treatment with si-DHRS4 induced cell cycle arrest and decreased the percentage of cells in the S phase, and no obvious change was observed in cells cotransfected with si-DHRS4 and miR-29a-5p inhibitor in both C6 and U87 lines (Figures 5C,D; *p* < 0.001 for both). The results of the CCK-8 assay were consistent with those of the Edu assay (Figures 5E,F).

Transwell chamber migration assays and statistical analyses also demonstrated that the migration capacity of C6 and U87 cells was reduced in response to si-DHRS4 transfection, whereas no changes were observed after cotransfection with miR-29a-5p inhibitor. Meanwhile, Transwell invasion assays and statistical analyses showed that the invasion capacity of C6 and U87 cells decreased in response to si-DHRS4 transfection, whereas no changes were observed after cotransfection with miR-29a-5p inhibitor (Figures 5G–J; *p* < 0.01, *p* < 0.001, respectively).

Wound healing assays and statistical analyses were performed to detect the motility capacity of C6 and U87 cells cotransfected with si-DHRS4 and miR-29a-5p inhibitor. The results showed that the motility capacity of C6 and U87 cell lines was decreased in cells transfected with si-DHRS4, whereas no changes were observed after cotransfection with miR-29a-5p inhibitor (Figures 5K,L; *p* < 0.05, *p* < 0.01, *p* < 0.001, respectively). These data suggested that DHRS4 has a tumor-promoting role in glioma cells through miR-29a-5p, and its knockdown rescues the down-regulation of proliferation, migration, and invasion in miR-29a-5p inhibitor/glioma cells *in vitro*.

### DHRS4 Stimulates Tumorigenicity of Glioma Cells *in vivo*

**FIGURE 6:**
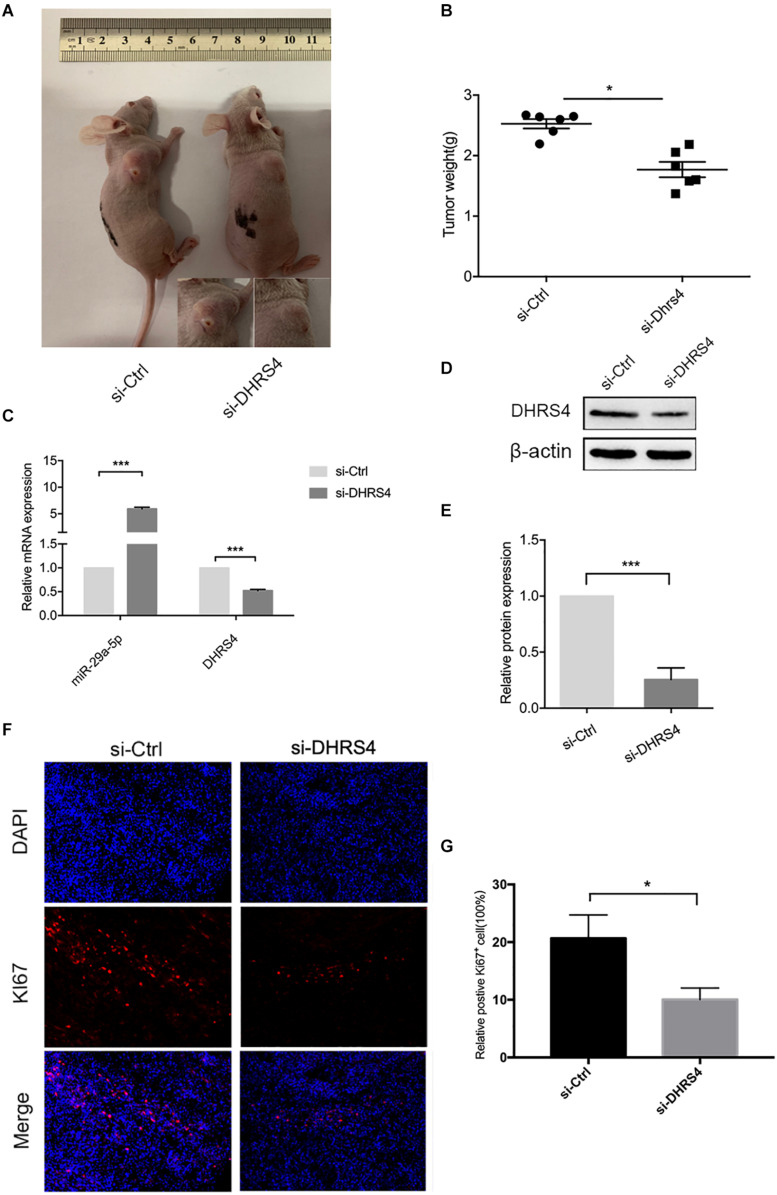
DHRS4 stimulates the tumorigenicity of glioma cells *in vivo*. **(A,B)** si-DHRS4 significantly inhibited the growth of xenografts formed, as compared with that in si-Ctrl group. **(C–E)** The mRNA and protein levels of miR-29-5p and DHRS4 in xenografts treated with si-DHRS4 and si-Ctrl group. **(F,G)** Representative photomicrographs of tumor sections after IF analysis (left panels) and data quantification (right panel) for Ki-67 expression. Scale bar, 100 μm, ***p < 0.001.

Having established that si-DHRS4 inhibits glioma cell proliferation, invasion, and migration *in vitro*, we evaluated the growth promoting effect of DHRS4 *in vivo* using a subcutaneous tumorigenesis experiment. We chose U87 cells for the *in vivo* model. Mice were subcutaneously injected with si-DHRS4, si-Ctrl (Figures 6A,B). Measurement of tumor weight showed that si-DHRS4 tumors grew at a significantly slower rate than si-Ctrl. RT-PCR and Western blotting demonstrated a significantly low expression of DHRS4 in si-DHRS4 tumors (Figures 6C–E; *p* < 0.001). In addition, Ki-67 staining showed that si-DHRS4 tumors had fewer proliferative cells than si-Ctrl group (Figures 6F,G; *p* < 0.001 for both). These results demonstrated that DHRS4 promotes the tumorigenesis of glioma cells *in vivo*, and further research is warranted to explore this effect.

## Discussion

Glioma is the most common primary brain tumor. Similar to other tumors, glioma mainly results from genetic risk factors and environmental carcinogenic factors (23). To date, molecular targeted therapy has been widespread. Traditional therapy affects cell damage and cytotoxicity, whereas molecular targeted therapy has the advantages of targeted specificity, stable effects, and corresponding regulatory functions and no cytotoxic effects (24–26). Glioma has been researched extensively, and studies have shown that miRNAs have emerged as key factors involved in several biological processes, including development, differentiation, cell proliferation, and tumorigenesis (27).

miR-29a is a conserved miRNA involved in the regulation of several coordinated posttranscriptional programs affecting different biological processes. For example, miR-29a represses the translation of multiple extracellular matrix proteins, and miR-29a depletion leads to fibrosis in several tissues (28). In previous studies, the Croce team found that the overexpression of mir-29a can slow the growth of leukemia cell lines, suggesting that mir-29a in chronic lymphocytic leukemia as a tumor suppressor gene. Park’s team, however, found that increasing circulating levels of mir-29a in mice led to the development of leukemia, suggesting that the miRNA could play a different role in the progression of cancer (29). Similarly, miR-29a regulates a complex program of cell growth and invasion in glioblastoma; decreased miR-29a expression is closely correlated with aggressive phenotypes and shorter survival times of glioma patients (21). In addition, a study has shown that miR-29a increases growth and invasion in glioblastoma; this program involves not only coactivation of the AKT/PI3K and Wnt pathways through the downregulation of PTEN and EphB3, but also the activation of a newly discovered Sox4/Hic5 invasion pathway (19). The above results show us the two sides of mi-29a. A further functional study has indicated that miR−29a−3p inhibits cell migration and invasion of a colorectal cancer cell line by suppressing CDC42BPA mRNA expression (30). In our other experiment, through gene sequencing, it was found that miR-29a-5p was highly expressed in neural stem cells and inhibited its differentiation into glial cells. Inspired by the above research and our previous studies, we boldly chose mi-29a-5p as the research object. In the present study, we confirmed that miR29a-5p was down-regulated in glioma cells, and treatment with an inhibitor of miR-29a-5p enhanced proliferation, migration, and invasion in C6 and U87 cells, whereas the mimic had the opposite effect *in vitro*. Therefore, the present results highlight the value of miR-29a-5p in glioma cells, although the mechanisms still need to be explored.

DHRS4 is an important metabolic enzyme in the human body, and it is expressed predominantly in the liver. DHRS4L1 and DHRS4L2 appeared in succession through DHRS4 gene duplication, and the latter is a recent member in genomic evolution. The DHRS4 gene cluster is located in an area of segmental duplications, and its expression is downregulated in certain human cancers (31). Recent data suggest that DHRS4-AS1 is a tumor inhibitor in clear cell renal cell carcinoma (ccRCC), demonstrating that DHRS4-AS1 is a potential prognostic biomarker in ccRCC (32). However, the role of DHRS4 in glioma has not been reported. The predictions of TargetScan and miRanda combined with our results indicated that DHRS4 is a target of miR-29a-5p. More crucially, through GEPIA analysis, we found that DHRS4 expression was significantly increased in human GBM tissues, as verified *in vitro* and *in vivo*. In addition, RT-PCR and Western blotting showed that miR-29a-5p expression was negatively correlated with DHRS4, thus implying a negative relationship between miR-29a-5p and DHRS4. We next continued to explore the relationship between miR-29a-5p and DHRS4. miRNAs negatively modulate target gene expression by directly binding to the corresponding 3’-UTR (33, 34). Our luciferase assays showed that miR-29a-5p directly regulated the 3’-UTR of DHRS4. The above experiments suggest that DHRS4 may play a key role in glioma as a direct target of miR-29a-5p. Our further investigations showed that knockdown of DHRS4 inhibited the proliferation, migration, and invasion of glioma cells *in vitro*. We additionally set up a rescue model and found no marked changes in glioma cell proliferation, migration, and invasion upon cotransfection of miR-29a-5p inhibitor and si-DHRS4, compared with si-Ctrl treatment alone, thus indicating that DHRS4 promotes the proliferation, invasion, and migration of glioma cells and is closely associated with the expression of miR-29a-5p. Moreover, we established a subcutaneous tumor model and confirmed that DHRS4 promotes tumor growth *in vivo*. Taken together, these findings provide insights into the mechanisms underlying the effect of miR-29a-5p on suppressing glioma progression, and DHRS4 may be directly involved as a potential cancer-promoting molecule. However, the detailed role and mechanism by which DHRS4 promotes glioma development and progression should be investigated in future work.

In summary, the results indicate that overexpression of miR-29a-5p suppresses glioma cell proliferation, invasion, and migration by directly targeting DHRS4. Furthermore, DHRS4 is considered an oncogene and has significant value as an unfavorable indicator for glioma patients, and it may serve as a therapeutic target in the future.

## Data Availability Statement

All datasets generated for this study are included in the article/Supplementary Material.

## Ethics Statement

The studies involving human participants were reviewed and approved by Changzheng Hospital, Second Military Medical University. The patients/participants provided their written informed consent to participate in this study. The animal study was reviewed and approved by School of Medicine, Nantong University. Written informed consent was obtained from the owners for the participation of their animals in this study.

## Author Contributions

YD and YZ conceived and designed this study and critically revised the article. YD and ZC performed the main experiments. WZ and GC assisted with the xenograft model construction in mice. ZW and XW assisted with the statistical analysis of data. HH assisted with specimen provision and analysis. YD drafted the manuscript and performed the literature review. All authors had final approval of the submitted versions.

## Conflict of Interest

The authors declare that the research was conducted in the absence of any commercial or financial relationships that could be construed as a potential conflict of interest.

## Footnotes

1
www.thermofisher.com

2
www.appliedbiosystems.com

3
http://www.Targetscan.org/

4
www.ribobio.com

## Supplementary Material

FIGURE S1 **(A)** Quantitative RT-PCR analyses of miR-29a-5p in C6 and U87 cells with miR-29a-5p mimics or inhibitor and the control cells in Figure 2, ^∗∗^*^∗^p* < 0.001.
