# Correction: miR-29a-5p regulates the proliferation, invasion, and migration of gliomas by targeting DHRS4

**DOI:** 10.3389/fonc.2025.1643256

**Published:** 2025-07-24

**Authors:** Yong Dai, Zhenhua Chen, Wei Zhao, Gang Cai, Zhifeng Wang, Xuejiang Wang, Hongkang Hu, Yi Zhang

**Affiliations:** ^1^ Department of Neurosurgery, Second Affiliated Hospital of Nantong University, Nantong, China; ^2^ Department of Neurosurgery, Changzheng Hospital, Second Military Medical University, Shanghai, China

**Keywords:** gliomas, miR-29a-5p, DHRS4, proliferation, invasion, migration

In the published article, there was an error in **Figure 2** and **Figure 5** as published. Due to an oversight in the preparation process of the image, two different parts of the data in each figure were accidentally overlaid, resulting in overlapping visuals. This led to an inaccurate representation of the experimental results. Also, in **Figure 2H**, the authors accidentally spliced the experimental results of U87 cells in the miR-29a-5p mimics group into the control inhibitor group (red box). The corrected **Figure 2** and its caption appears below.

**Figure 2:**
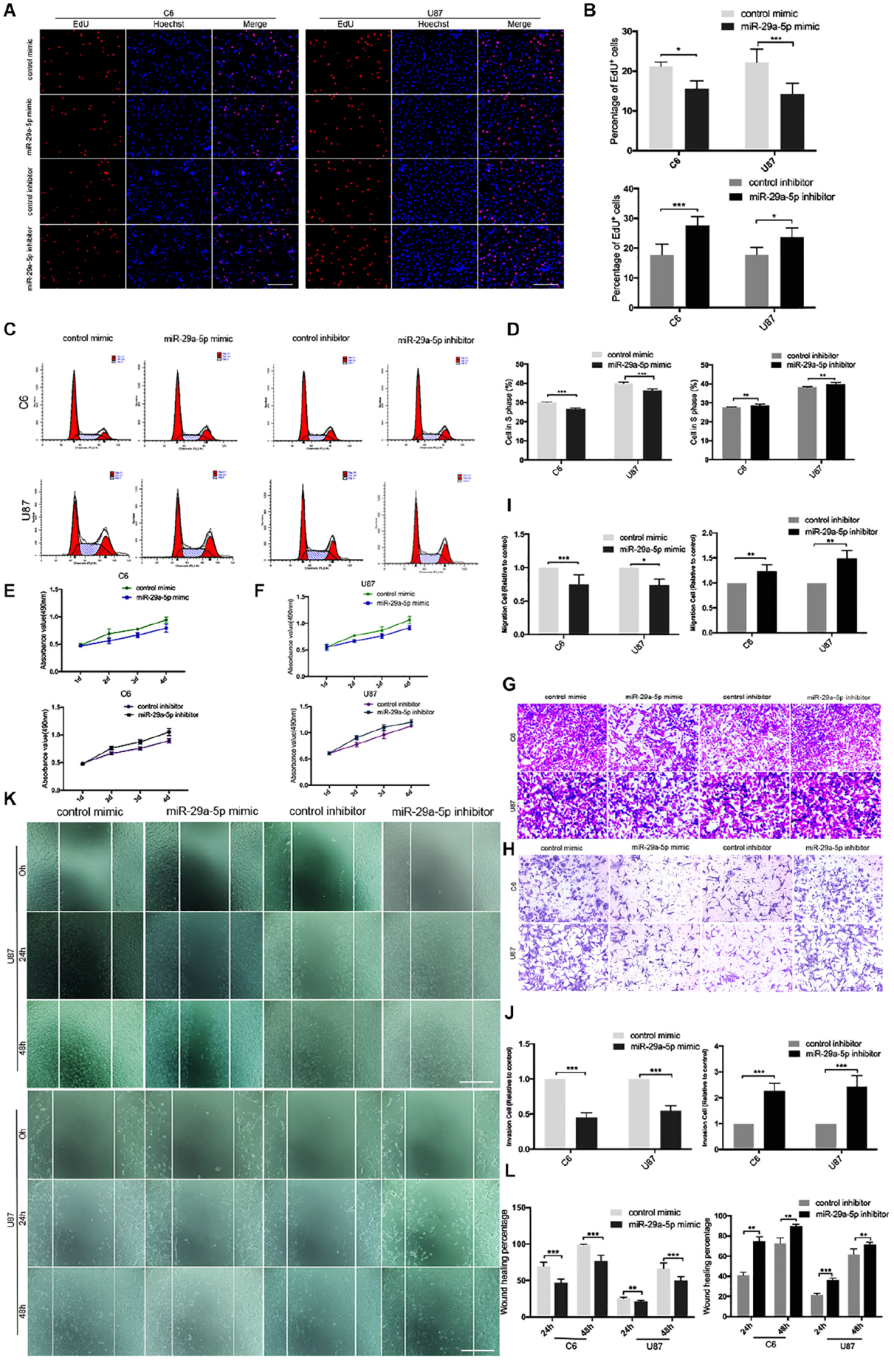
miR-29a-5p overexpression decreases glioma cell proliferation, invasion, and migration. **(A, B)** Fluorescence micrographs (left panels) and data quantification (right panel) from BrdU proliferation assays used to investigate the effects of miR-29a-5p upregulation and downregulation on BrdU (red) incorporation into nuclei (DAPI, blue) in C6 and U87 cells. Data shown in the right panel are mean ± SEM, *p < 0.05, ***p < 0.001, unpaired t test. Scale, approximately 100 μm. **(C, D)** Flow cytometry was used to assess the cell cycle distribution of C6 and U87 cells transfected with miR-29a-5p mimics and inhibitor or the control cells for 24 h and stained with PI (left panels). Representative and quantitative results for the S phase are shown (right panel) **p < 0.01, ***p < 0.001. **(E, F)** Cell proliferation was measured by CCK-8 analysis starting 1 day after transfection with miR-29a-5p mimics and inhibitor or the control cells in C6 and U87 cells every day. **(G, I)** Transwell assays of the migration in C6 and U87 cells transfected with miR-29a-5p mimics and miR-29a-5p inhibitor or the control cells, Representative and quantitative results for migration are shown. Columns are the averages of three independent experiments, *p < 0.05, **p < 0.01, ***p < 0.001. **(H, J)** Transwell assays of the invasion in C6 and U87 cells transfected with miR-29a-5p mimics and miR-29a-5p inhibitor or the control cells; invasion of the above cells was quantitatively analyzed. Columns are the averages of three independent experiments, *p < 0.05, **p < 0.01, ***p < 0.001. **(K, L)** Wound healing assays revealing wound closure with miR-29a-5p mimics and miR-29a-5p inhibitor or the control cells in C6 and U87 cells at 0-, 24-, and 48–h timepoints after transfection. Columns are the averages of three independent experiments, **p < 0.01, ***p < 0.001.

**Figure 5:**
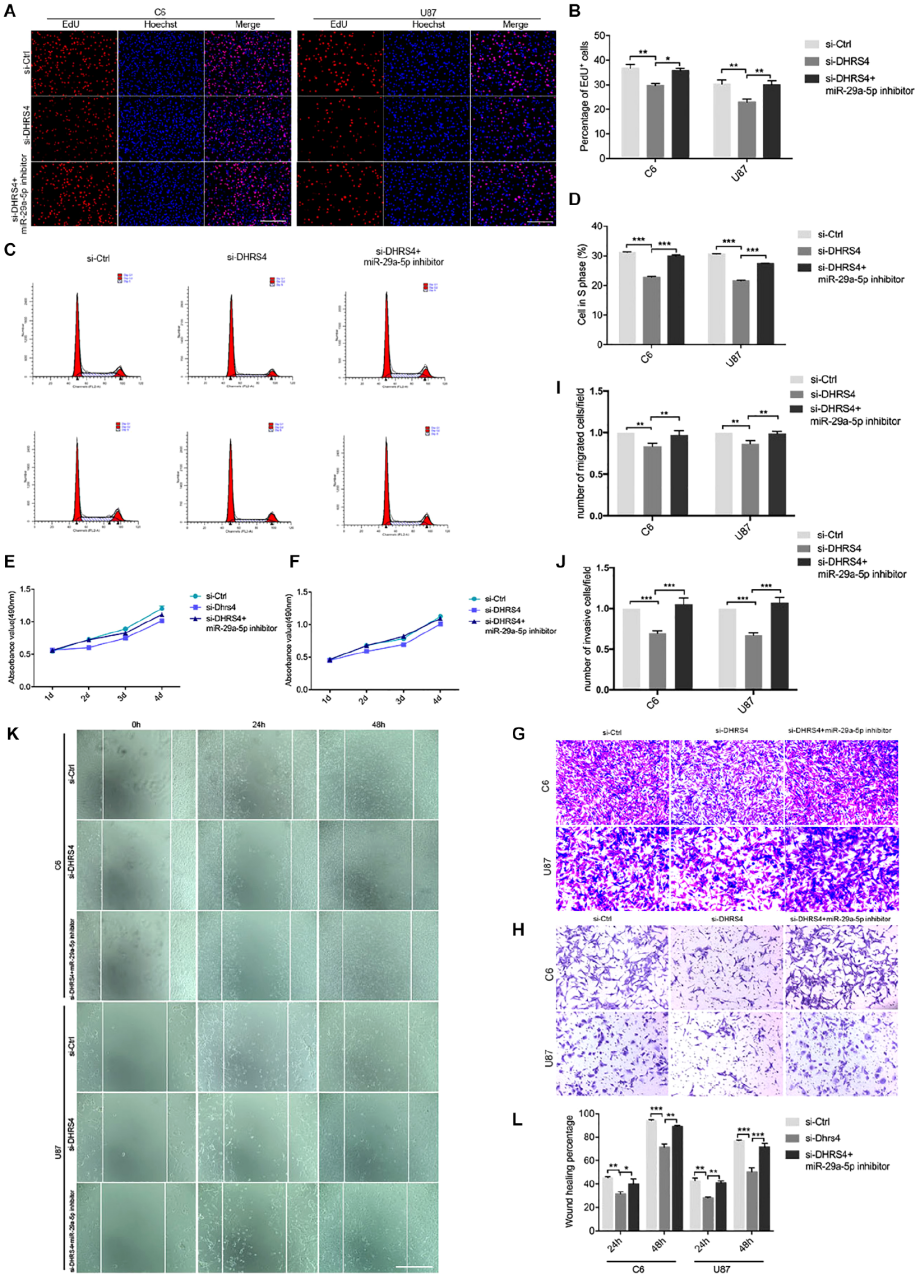
Knockdown of DHRS4 inhibits glioma cell proliferation, invasion, and migration. **(A, B)** Fluorescence micrographs (left panel) and quantification (right panel) of BrdU proliferation assay data for si-Ctrl, si-DHRS4, or si-DHRS4 plus miR-29-5p in C6 and U87 cells. Data shown in the right panel are mean ± SEM, *p < 0.05, **p < 0.01, unpaired t test. Scale, approximately 100 μm. **(C, D)** Flow cytometry was used to assess the cell cycle distribution of C6 and U87 cells transfected with si-Ctrl, si-Dhrs4, or si-DHRS4 plus miR-29-5p inhibitor for 24 h and stained with PI (left panels). Representative and quantitative results of S phase are shown (right panel), ***p < 0.001. **(E, F)** Cell proliferation from the above transfected cells was measured with CCK-8 analysis. **(G, I)** Transwell assays of the migration in C6 and U87 cells with si-Ctrl, si-DHRS4, or si-DHRS4 plus miR-29-5p inhibitor. Representative and quantitative results for migration are shown. Columns are the averages of three independent experiments, **p < 0.01. **(H, J)** Transwell assays of the invasion in C6 and U87 cells with si-Ctrl, si-DHRS4, or si-DHRS4 plus miR-29-5p inhibitor. Representative and quantitative results for invasion are shown. Columns are the averages of three independent experiments, ***p < 0.001. **(K, L)** Wound healing assay results revealed wound closure of C6 and U87 with si-Ctrl, si-DHRS4, or si-DHRS4 plus miR-29-5p inhibitor. Columns are the averages of three independent experiments, *p < 0.05; **p < 0.01, ***p < 0.001.

In the published article, there was an error in **Figure 5** as published. Due to an oversight in the preparation process of the image, two different parts of the data in each figure were accidentally overlaid, resulting in overlapping visuals. This led to an inaccurate representation of the experimental results. Also, in **Figure 5G**, an experimental picture from the si-Ctrl group was mistakenly spliced into the si-DHRS4+miR-29-5p inhibitor group (blue box). The corrected **Figure 5** and its caption appears below.

The authors apologize for these errors and state that this does not change the scientific conclusions of the article in any way. The original article has been updated.

