# Supplementary figures and images for: miR-29a-5p Regulates the Proliferation, Invasion, and Migration of Gliomas by Targeting DHRS4
Source: Front Oncol. 2020 Sep 10;10:1772. doi: 10.3389/fonc.2020.01772 (PMC7511594; doi:10.3389/fonc.2020.01772)

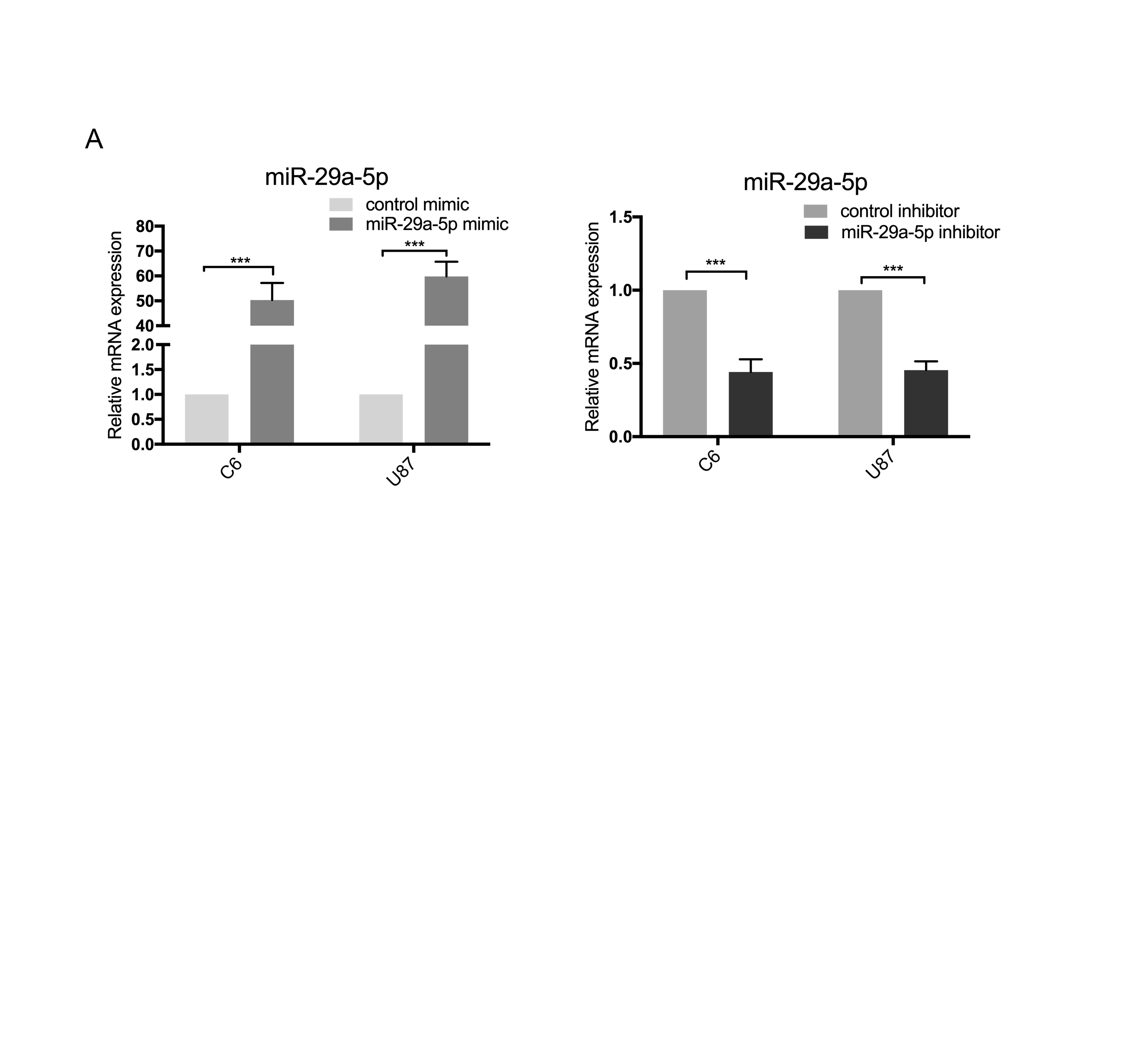

Supplement: FIGURE S1 — (A) Quantitative RT-PCR analyses of miR-29a-5p in C6 and U87 cells with miR-29a-5p mimics or inhibitor and the control cells in Figure 2, ∗∗∗p < 0.001. [file Image_1.TIF]
